# Managing the moral expansion of medicine

**DOI:** 10.1186/s12910-022-00836-2

**Published:** 2022-09-22

**Authors:** Bjørn Hofmann

**Affiliations:** 1 Institute for the Health Sciences, The Norwegian University of Science and Technology (NTNU), PO Box 191, 2802 Gjøvik, Norway; 2 Centre of Medical Ethics, University of Oslo, PO Box 1130, N-0318 Oslo, Norway

**Keywords:** Disease, Health, Wellbeing, Expansion, Overdiagnosis, Medicalization, Overtreatment

## Abstract

Science and technology have vastly expanded the realm of medicine. The numbers of and knowledge about diseases has greatly increased, and we can help more people in many more ways than ever before. At the same time, the extensive expansion has also augmented harms, professional responsibility, and ethical concerns. While these challenges have been studied from a wide range of perspectives, the problems prevail. This article adds value to previous analyses by identifying how the moral imperative of medicine has expanded in three ways: (1) from targeting experienced phenomena, such as pain and suffering, to non-experienced phenomena (paraclinical signs and indicators); (2) from addressing present pain to potential future suffering; and (3) from reducing negative wellbeing (pain and suffering) to promoting positive wellbeing. These expansions create and aggravate problems in medicine: medicalization, overdiagnosis, overtreatment, risk aversion, stigmatization, and healthism. Moreover, they threaten to infringe ethical principles, to distract attention and responsibility from other competent agents and institutions, to enhance the power and responsibility of professionals, and to change the professional-beneficiary relationship. In order to find ways to manage the moral expansion of medicine, four traditional ways of setting limits are analyzed and dismissed. However, basic asymmetries in ethics suggest that it is more justified to address people’s negative wellbeing (pain and suffering) than their positive wellbeing. Moreover, differences in epistemology, indicate that it is less uncertain to address present pain and suffering than future wellbeing and happiness. Based on these insights the article concludes that the moral imperative of medicine has a gradient from pain and suffering to wellbeing and happiness, and from the present to the future. Hence, in general present pain and suffering have normative priority over future positive wellbeing.

## Background

Medicine has become ever more encompassing, moving from 2400 diseases in Sauvage’s ”Nosologia methodica” in 1768 [1] to more than 55,000 codes in ICD-11 [2]. Moreover, medical taxonomies have expanded from classifications of causes of *death*, *diseases*, and *injuries* to conditions related to *health*. For example, the International Classification of Diseases has added “Related Health Problems” to its name and content. Accordingly, health professionals are expected to address ever broader determinants of health and social issues [3].

Certainly, this expansion of medicine is a result of extended knowledge and disease differentiation and has many good intentions and implications. However, it also has a wide range of negative effects, such as overdiagnosis [4, 5], overtreatment [6], medicalization [7–9], low-value care [10–14], risk aversion, hype [15] and hubris [16]. Moreover, it generates ever larger costs for individuals and societies [14].

Accordingly, many measures have been initiated to curb the unwarranted expansion of medicine, such as NICE’s DoNotDo Database (2006), the Choosing Wisely campaign (2012), Slow Medicine (2013), Too Much Medicine (2013), Preventing Overdiagnosis (2013), Lown Institute’s Right Care Movement (2013), and the need to take action to protect individuals from medical interventions that are likely to cause more harm than good [17], i.e., quarternary prevention (1995/2015).

Correspondingly, a wide range of theoretical approaches have been applied to analyze and address the downsides of the vast expansion of medicine, which has been studied as a surge in knowledge [18], extensive emergence of technologies [19], expansion of power [20], cultural extension [21] or culmination [22], the combination of power and knowledge [23] etc. Despite these efforts the negative effects of the expansion are prevailing. One reason for this may be that we have focused too much on the characteristics of the expansion and too little on its root causes – too much on its *moral effects* and too little on its *moral origin*.

Therefore, it is important to investigate the vast expansion of medicine as a moral expansion: the expectations and aspirations to *do more good*. Given the tremendous progress of medicine it seems paradoxical that medicine is doing better while the population is feeling worse [24, 25], and that the overall outcomes are *bad* from doing more of what is considered to be *good* [25]. Hence, analyzing the vast expansion of medicine in terms of an *expansion of medicine’s moral imperative* can achieve new insights and new measures to ascertain good expansion.

For millennia, the moral foundation of medicine has been an imperative to help people, and the drive and target of this imperative has been people’s *pain* and *suffering* [26]. The pain (dis-ease) of a person has urged health professionals to develop and use their knowledge and skills to reduce the pain and alleviate the suffering by amending the condition and prognosticate its course. The traditional target of this imperative has been to define, detect, and treat disease, or otherwise to mitigate its consequences. Certainly, vast advances in science and technology have facilitated this development. However, at the basis of these processes is a threefold expansion of the moral foundation of medicine:

1. *Expanding disease beyond pain and suffering*: Medicine has extended what is considered to be bad (and subject to a moral imperative) from experienced *pain* and *suffering* to observed indicators (biomarkers), risk factors (hypertension), social phenomena (grief), aesthetic phenomena (protruding ears) and various non-harmful conditions [27]. While the benefits of this *expansion of morally relevant phenomena* to include in the imperative are ample, the main downside is unnecessary labelling and treating people as “diseased” without necessarily reducing their pain or suffering.
2. *From present to future pain and suffering*: Medicine has expanded its moral imperative from addressing concrete contemporary pain and suffering to potential pain and suffering in the future. No doubt, by pre-empting disease in prevention, pain and suffering will be avoided. However, this *temporal expansion from present to future* also has significant side-effects: health anxiety, potential unnecessary diagnostics and treatment, and elevating health to a super value (healthism) [28].
3. *From reducing negative wellbeing to promoting positive wellbeing*: The focus of medicine has expanded from negative experiences, such as pain and suffering, to positive experiences, such as pleasure and happiness. While improving positive wellbeing obviously is good, making this a primary purpose of medicine is challenging, e.g., because it can be more difficult to define positive than negative wellbeing and to increase the positive wellbeing of healthy persons than to reduce the negative wellbeing in terms of pain and suffering. Given limited resources and the many people who suffer from disease, this *expansion of wellbeing* calls for ethical reflection.

Hence, while the vast expansions of medicine boost its benefits as more diseases are defined, differentiated, detected, and treated, it also expands the potential harms. Accordingly, the key question becomes *how can we manage the expansion of the moral imperative in medicine in order to obtain the benefits and avoid the harms*?

To address this question each expansion will be analyzed, together with its challenges, and the corresponding ethical issues. Given these challenges the article will investigate four traditional approaches to manage the moral expansion of medicine, such as (a) aligning medicine with its basic concepts (disease, therapy, and naturalness), (b) to bring medicine in accordance with its ethos, (c) to make medicine adhere to its basic goals, and (d) in terms of making medicine better.

As these approaches may not give sufficient normative guidance, the article will assess what it means to make medicine *better*. In particular, it will investigate a basic asymmetry in ethics suggesting that it is more justified to define and address negative wellbeing, such as pain and suffering than positive wellbeing, such as pleasure. This will be combined with an analysis of differences in epistemology, especially whether present negative wellbeing represent less uncertainty than future positive wellbeing.

Based on this analysis it will be concluded that the moral imperative of medicine has a gradient from pain and suffering to (positive) wellbeing and happiness, and from present to future.

## Main text

### Three types of expansions, their problems, and potential solutions

#### Expanding disease beyond pain and suffering

**Fig. 1:**
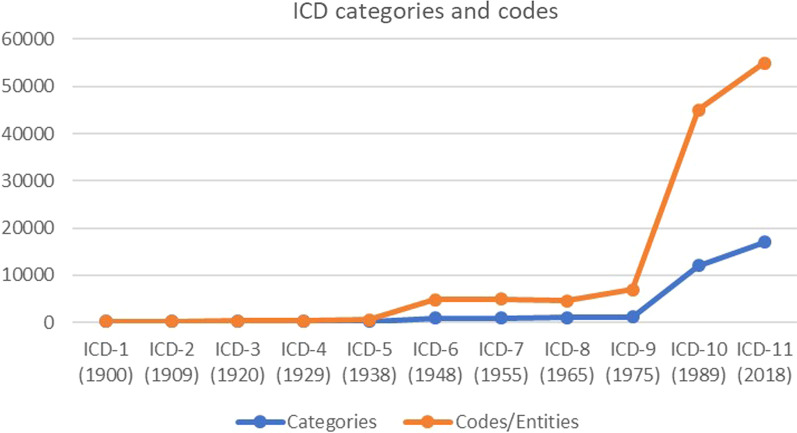
The expansion in number of disease categories and codes from ICD-1 in 1900 till ICD-11 in 2018. Expanded from [32].

 The first expansion of medicine’s moral imperative has been through the extension of the phenomena that medicine is targeting: from experienced phenomena, such as pain and suffering, to non-experienced phenomena, such as paraclinical signs and indicators. This has mainly happened by a vast expansion of the phenomena that fall under the concept of disease (and therefore are considered to be *bad*). From John Graunt’s Bills of Mortality in 1665 till ICD-11 [2], DSM [29], ICPC [30], and in ICF [31] more people are diagnosed with ever more diseases. Figure 1 shows the expansion of number of disease categories and codes from ICD-1 in 1900 till ICD-11 in 2018.

Part of this expansion is because we know more about bodily and mental mechanisms than ever before. By differentiating existing diseases in more precise and actionable entities, more people can be helped—better and earlier than ever before.

However, our causal and predictive powers are (still) less developed than our identifying skills. We tend to infer from discovering conditions to the imperative of handling them [16, 33]. We can detect many more precursors of disease than ever before [34], but we still lack knowledge of whether they will develop into what is morally relevant [27]. Hence, we appear to be better at detecting than at predicting [32]. This results in expanded disease labelling (and potential anxiety), unnecessary subsequent diagnostics and treatment, i.e., overuse of health care services [35, 36].

To a large extent medicine has expanded its subject matter from manifest disease to indicators, for example by labelling indolent conditions as disease [5] or labelling predictors or precursors “disease,” such as pre-diabetes and pre-Alzheimer. Moreover, risk factors, such as hypertension and obesity have been classified as disease [37–42]. Additionally, species-typical characteristics, such as menopause and aging, are made disease [43]. The process where preclinical non-symptomatic conditions are understood to be diseases has been called *diseasisation* [44].

Common to the expansion of disease by including indicators, predictors, precursors, and risk factors into our conceptions of disease is that they can result in overdiagnosis and overtreatment [45], health anxiety, and that we may do more harm than good [46, 47].

However, the expansion of the moral imperative of medicine through the expansion of the concept of disease (as something bad) can be seen in other fields as well. For example, many aesthetic phenomena and non-harmful conditions, such as protruding ears and funnel chest (pectus excavatum) have been classified and handled as diseases even in cases where there is no functional reduction (in hearing or breathing). While socially helpful, it is not clear that doing so reduces pain and suffering in individuals or in society at large [48].

Medicine has also been heavily criticized for including ordinary life experiences and social phenomena in its subject matter, i.e., for medicalization [9, 28, 49]. Grief [50, 51], sexual orientation [52, 53] or identity [54, 55], social behavior (ADHD) [56], and love [57] are but a few examples. While medicalization certainly can be positive [58, 59], it can also divert responsibility from other actors and institutions that may be better at handling these phenomena. For example, families and social networks may sometimes be better at handling grief than health care.

Another important development is that classification systems have been expanded to include health-related issues, not considered to be disease. The reason for this is mainly pragmatic, as classified conditions give rights to attention and care. For some conditions (such as obesity) organizations explicitly state that they do not consider their condition to be disease but in order to obtain access to health services, they insist that it should be classified as a disease [60, 61].

Another *pragmatic* expansion of disease is when a condition is made disease because it can be detected and treated [62]. Erectile dysfunction (due to the discovery of sildenafil) is but one example. This relates to *disease mongering*, i.e., making biological or social conditions disease in order to sell diagnostic tests or therapies. Low testosterone (Low T) is one example of this [63, 64]. The problems with this are manifold: healthy persons are made patients, it results in anxiety, overtreatment, or negative side-effects.

The various types of expansion of the concept of disease that contribute to the expansion of medicine’s moral imperative are summarized in Table 1. The main challenges with this type of expansion of the moral imperative of medicine are potential harm from unnecessary diagnostics or treatment, overdiagnosis, overtreatment [5, 65], stigmatization, health anxiety, medicalization, and low-quality and low-value care [13, 14, 66]. Moreover, the allocation of resources for persons without pain and suffering raises concern about equity and justice (opportunity costs with respect to those who are in pain and are suffering). Moreover, the increased involvement with ever larger parts of people’s lives raises concern of professional power and responsibility, as well as professional integrity. Additionally, it changes the professional-beneficiary relationship, as health professionals approach people who do not know that they need help instead of people calling on professionals for help.

Each of these ethical challenges warrant specific analysis. Here the point is that medicine has expanded what falls under the concept (and classification) of disease, and thus counts as the subject matter for the moral imperative of medicine. It has done so by addressing phenomena that cannot be directly related to people’s experience of pain and suffering. This expansion of the moral imperative of medicine to a wide range of phenomena that are not closely linked to experienced pain and suffering may become ever more pertinent in the future when an unprecedented number of new biomarkers, risk factors, social issues, and indicators emerge from the convergence of omics, Big Data, Artificial Intelligence, precision medicine [67], and enormous investments [68].

**Table 1:** Seven types of expansion of disease with descriptions, examples and potential challenges. Adapted and expanded from [48]

Type of expansion	Description	Example	Problem/challenge
Medicalization (expansion of experienced phenomena)	Including ordinary life experiences [9]	Grief, sexual orientation (homosexuality)	Inefficient or inappropriate handling
Overdiagnosis (expansion of non-experienced phenomena)	Labelling indolent conditions as disease [5]	Ductal carcinoma in situ (DCIS)	Prognostic uncertainty [27], overtreatment [45]
Aesthetic expansion	Treating aesthetic characteristics as disease [69]	Protruding ears	Reinforcing or enhancing attitudes and stigma
Pragmatic expansion	Making something disease because it can be detected and treated [62]	Erectile dysfunction, “alcoholism,” hypertension	Making healthy persons patients, overtreatment, side-effects
Conceptual expansion	Expanding definitions or indications of disease [43]	Pre-diabetes, pre-Alzheimer, (making menopause or aging a disease)	Making healthy persons patients, overtreatment, side-effects
Ethical expansion	Making something disease because that will provide attention and access to care	Obesity [60], Attention Deficit Hyperactivity Disorder (ADHD), gender incongruencen [55]	Pathologization, stigmatization, opportunity costs
Disease mongering	Making biological or social conditions disease in order to sell diagnostic tests or therapies	Low testosterone (Low T) [63, 64], “restless legs syndrome” [70]	Making people patients for the purpose of profit

Hence, when expanding the moral imperative to phenomena and conditions that can be closely connected to people’s experiences, such as pain and suffering, the expansion is warranted. However, when targeting conditions that will not result in pain and suffering (overdiagnosis), where the pain or suffering is an ordinary-life experience or socially constructed (medicalization), or where making something a disease will increase stigmatization, medicine makes a problematic moral expansion that raises profound ethical concerns as it does more harm than good.

#### From present to future pain and suffering

The second expansion of the moral imperative has been to extend medicine’s concern for reducing negative wellbeing from the present to the future: from alleviating current pain and suffering to avoiding this in the future. While preventive medicine measures are directed towards the traditional target of medicine’s moral imperative, i.e., the phenomena of pain and suffering, they tend to target healthy people to avoid such phenomena in the future. The idiom “an ounce of prevention is worth a pound of cure” expresses well the rationale behind this expansion.

While it has been argued that preventive measures are tasks beyond the scope of medicine [28, 71], in practice it is clearly one of its integrated and time consuming tasks [72]. Moreover, while some preventive measures are effective in avoiding and reducing pain and suffering, other measures, such as screening and health checks, are shown to be less effective than presumed [73–77]. Other institutions than the health services may be more important for preventing disease. Alzheimer disease may serve as one example, where non-medical factors are pivotal for prevention [78].

Additionally, providing health services to presently healthy persons to avoid potentially future pain at the expenses of attention to persons with present pain and suffering, poses challenges in priority setting [79, 80]. Correspondingly, medicine has been criticized for exercising power over people’s private and ordinary life by various preventive measures and for changing the professional-patient relationship [81]. Introducing and enhancing health anxiety, risk aversion, orthorexia [82] and promoting healthism (elevating health to a super value) [28] have been shown to reduce the benefits of preventive measures.

In sum, expanding from present to future harm and suffering can do more harm than good, distract from other more appropriate measures, challenge justice, displace power and responsibility, and challenge the patient-professional relationship. This does of course not rule out preventive measures, which are clearly warranted, for example when they in fact obstruct future pain and suffering without opportunity costs for presently suffering persons that could be helped. However, and adding to the problem, we do not always know that the positive future effects will occur and will outweigh present and future negative effects. See below.

**Fig. 2:**
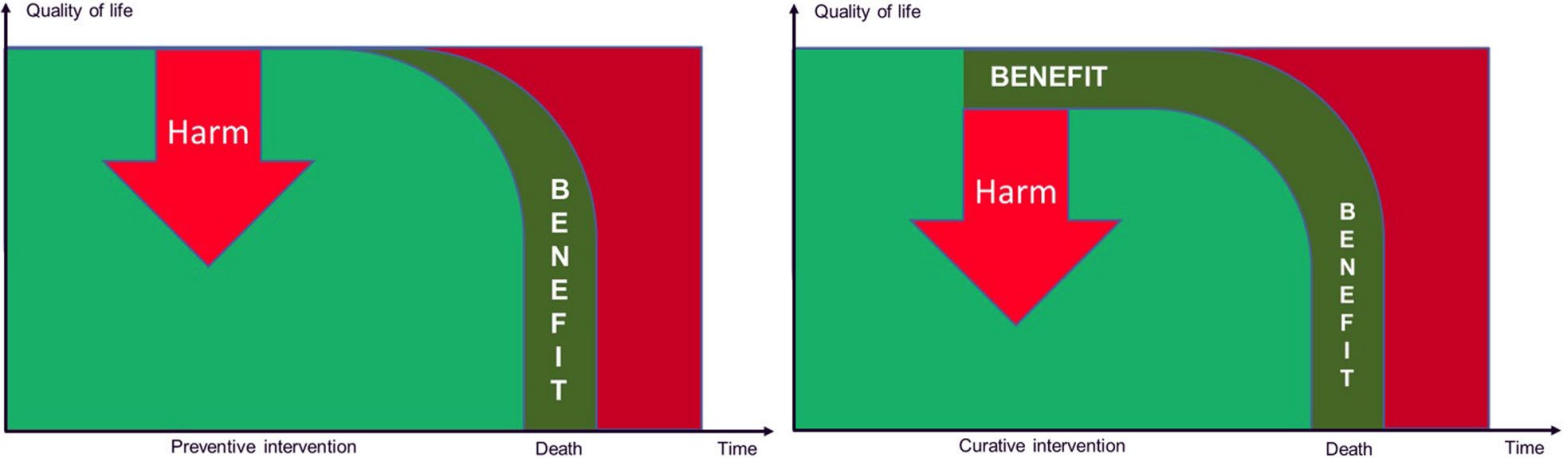
Comparing harms and benefits in curative and preventive measures

 Figure 2 illustrates the dilemma of balancing present and future harms and benefits with curative versus preventive measures. For curative measures the benefits and the harms often appear at the same time. For preventive measures, the benefits may occur in the future, while the harms can occur at present.

Hence, the very reasonable *temporal moral expansion from present to future* pain and suffering has some important premises and potential negative effects. Accordingly, we need to address these issues in order to ascertain the benefits of preventive medicine and avoiding its harms.

#### From alleviating pain to promoting pleasure

The third moral expansion of medicine has been to extend its imperative from addressing negative wellbeing to positive wellbeing, e.g., from pain to pleasure. In line with WHO’s definition of health as “a state of complete physical, mental and social well-being and not merely the absence of disease or infirmity” [83], positive wellbeing has been promoted as the goal of medicine and health policy [84]. No doubt, medicine has obtained great success as “the greatest benefit to mankind ”[18]. Moreover, its recipe for improving the conditions for humans has inspired a range of other fields, such as social care. Accordingly, the normative goal of medicine has expanded beyond the traditional end to alleviate and avoid pain and suffering. It has become to increase the positive wellbeing of human beings [85]. This may be strived for in many ways, e.g., by paying attention to the social determinants of health [86], by “improving” appearance, such as in cosmetic plastic surgery; by enhancing human characteristics, such as resilience, physical strength, sexual performance, gender identity, self-confidence [87], intelligence, emotional stability, longevity, moral capacity [88], love [57], and happiness [89]; or by fulfilling people’s wishes [90].

Certainly, to pay attention to positive wellbeing (as well as happiness and determinants of health) is crucial, but this may also come with some problems. For example, the *expansion of wellbeing* can absolve other institutions and politicians from their responsibilities for peoples’ positive wellbeing, i.e., it can become a moral distraction [3]. Moreover, emerging biotechnologies, such as personalized medicine, gene editing, and artificial intelligence, may be forceful tools to promote human positive wellbeing. However, given limited resources, it is contested whether positive wellbeing (e.g., happiness) should be the primary goal of medicine [85, 91, 92]. As there still are so many individuals with pain and suffering that can be addressed by healthcare, it can infringe the principle of justice to promote the positive wellbeing of a selected group of persons who are considered to be healthy.

Correspondingly, the expansion from alleviating pain to promoting pleasure also raises concerns for non-maleficence and beneficence as the consequences of promoting positive wellbeing are difficult to predict and measure and can be harmful [93]. As alluded to, making medicine the master of positive wellbeing may give it power over areas that are otherwise considered to belong to the realm of politics. When health professionals do not only help people with their pain and suffering, but are accountable for their wellbeing and happiness, it alters the professional-beneficiary relationship. The point here is not to dismiss medicine’s potential role in improving positive wellbeing but only to point out that this expansion raises specific moral concerns.

**Fig. 3:**
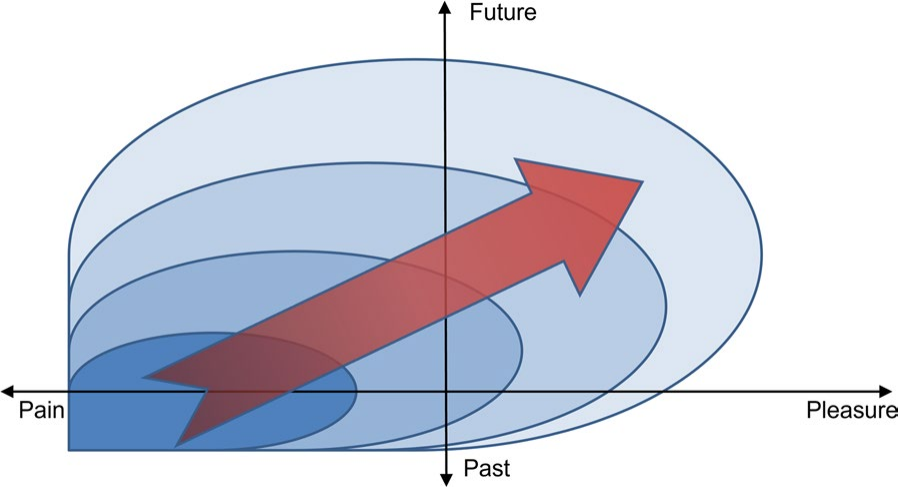
Moral expansion from pain to pleasure and from present/past to future

Hence, the expansion in the moral imperative from reducing negative wellbeing to promoting positive wellbeing raises concern for harm, beneficence, distraction, justice, power and responsibility, integrity, as well as altered relationship between professionals and beneficiaries. Figure 3 illustrates the moral expansion from pain to pleasure (expansion of wellbeing) and from present/past to future pain (temporal expansion).

### Managing moral expansions

So far, I have identified three types of expansions of the moral imperative in medicine: (1) by including a wide range of non-experienced phenomena beyond experienced pain and suffering; (2) including future benefits in addition to present harms; and (3) targeting people’s positive wellbeing. I have also pointed out potential problems with these expansions, such as overdiagnosis, overtreatment, medicalization, stigmatization, risk aversion, health anxiety, and healthism. Moreover, such implications can infringe basic ethical principles, such as non-maleficence, beneficence, and justice, and, to the extent that people are not informed, also autonomy. They can distract attention and responsibility from other competent agents (such as politicians), enhance the power and responsibility of professionals, and change the professional-beneficiary relationship.

These findings urge the question of how to address the moral expansions of the moral imperative of medicine. Let me briefly address the question along four traditional lines of thought: Aligning medicine with its (a) basic concepts, (b) ethos, (c) basic goals, and (d) with conceptions of making medicine better.

#### Aligning medicine with its basic concepts

The first approach to manage the expansion of the moral imperative of medicine would be to use *key concepts* in medical tradition, such as disease, therapy, and naturalness, as its normative measure. According to such approaches, medicine should stick to the treatment of disease (and abstain from enhancement of health) in order to (re-)establish natural human functioning (and abstain from improvement). However, naturalness can mean many things [94–96] and reference to nature may not provide robust normative guidance [97, 98]. The same problem occurs when we try to use the therapy-enhancement distinction to restrict the moral expansion of medicine. Vaccines are good counterexamples reducing the usefulness of this distinction, which has been shown to be blurred and not very action-guiding [97, 98].

Referring to the distinction between health and disease does not do any heavy lifting either, as the moral expansion undermines this traditional distinction [99]. As demonstrated above, the concept of disease is under continuous expansion. Moreover, it has been argued that concepts like health and disease are vague [100–103], fuzzy [104, 105], unnecessary [106, 107], or essentially contested concepts [108]. Hence, the basic concepts in medicine do not seem to do the job.

#### Aligning medicine with its ethos

Another approach to harness or manage the moral expansion of medicine could be to refer to *the ethos of medicine*, which has been defined as its “essential operative values”[109]. Accordingly, any moral expansion of medicine beyond its ethos is not warranted [110, 111]. However, the norms and values of medicine are diverse and changing. Moreover, there is no agreement on a consistent definition of ethos [109, 112–114], and referring to specific definitions of the ethos of medicine may not solve the problem either. For example, the ethos of medicine has been defined as “the normative social structure, moral shape or order” of a particular field (medicine) [110]. As the “moral shape or order” is exactly what is changing in the moral expansion described above, referring to the ethos of medicine does not seem very helpful for managing the moral expansion.

#### Aligning medicine with its basic goals

Yet another approach to control and direct the moral expansion is to assure that medicine is aligned with its goals. In their seminal work, Hanson and Callahan listed four basic goals of medicine: (1) prevention of disease and promotion of health, (2) relief of suffering (caused by malady), (3) care of the ill, and (4) avoidance of premature death [115]. However, there has been a substantial and extensive debate on what are the appropriate goals of medicine without culminating in consensus [115–128]. Additionally, there are many interpretations of each goal. For example, many of the examples of harmful expansion above are developed in accordance with the goals stated by Hanson and Callahan and colleagues. Biomarker testing and cancer screening resulting in overdiagnosis and overtreatment are but two examples. Moreover, new goals that expand the moral imperative of medicine have been suggested. For example, various kinds of welfarist goals of medicine [129–131] tend to augment rather than to control the moral expansion. The same problem occurs for attempts to define the “nature of medicine” [126, 132, 133], “the essence of medicine” [134], or “the end of medicine” (being different from the goal) [132]. Addressing all such approaches is beyond the scope of this article. The point here is rather that such teleological approaches may not have the norm-regulating impact that is required.

While acknowledging the difficulties with restricting or directing the expansion of the moral imperative of medicine as described above, there still seem to be some relevant options related to the concept of *goodness*.

#### Making medicine better

We can ask whether the moral expansion makes medicine *better*. In cases of overdiagnosis, overtreatment, stigmatization, and the creation of health anxiety the answer is no. However, the three types of moral expansions identified do not only have bad implications. They can certainly be helpful and even lifesaving, for example when avoiding a mortal disease by an identifying biomarker. The problem is to differentiate the good from the bad. It is partly a prognostic and predictive problem (see below) and partly a problem of knowing what we mean by *better.* If we can ascertain that a specific kind of moral expansion makes the life of patients or persons better, it should certainly be endorsed. However, as we have learned from the enhancement debate, which partly has confused quantity (more functioning) with quality (better life), it is not clear what makes the life of patients and persons better [97]. As there are many conceptions of the goals and values of medicine, there are many conceptions of what is better in medicine [128].

This indicates that the traditional ways of addressing moral issues in medicine (aligning medicine with its basic concepts, with its ethos, with its basic goals, and with conceptions of making medicine better) may not work for addressing the expansion of the moral imperative in medicine. Does this mean that there are no resources for managing the expansion of the moral imperative of medicine? Before concluding pessimistically on this question, let us briefly examine two crucial issues: differences in uncertainty and in moral imperative.

### Epistemic differences

Medicine is inevitably an uncertain endeavor. Diagnoses are inaccurate, prognoses are unreliable, and predictions and the outcomes of treatments are uncertain [135]. As already pointed out, our predictive powers are (still) less developed than our detecting skills. We are good at identifying indicators, but not so good at knowing whether they matter [136].

While uncertainty is existing both in present and with respect to the future, we know in general less about the future than about the present. Prognostic (and predictive) uncertainty adds to present uncertainty [27]. Hence, our understanding of good and bad here and now appears in general to be less uncertain than that in the future. Accordingly, it can be argued that overall present events have an epistemic advantage over future events, and that we should prioritize to promote good and avoid bad here and now compared to in the future. This is, however, not to say that a present uncertain good should have priority over a future uncertain good. The basic principle is epistemic, and not temporal.

Therefore, from an epistemic point of view (in cases where future events and outcomes are more uncertain than present events and outcomes) it can be argued that we should give priority to present events and outcomes. Such an argument could be made from the principle of beneficence (what is best for persons), from the perspective of maximizing utility (utilitarianism), from the duty to help individuals (deontology), and from several relational perspectives (virtue ethics). This is not the place to investigate all these perspectives. Suffice it here to indicate that the epistemic difference between present and future events can give priority to present events, and thus can provide resources to manage temporal expansions of the moral imperative of medicine.

### Asymmetries in ethics

Correspondingly, there may be sources for harnessing the expansion from negative to positive wellbeing. The moral philosopher Knut Erik Tranøy has argued that negative notions, such as bad, disease, and pain are logically more fundamental and definite than positive notions, such as good, health, and pleasure [137]. According to Tranøy negative notions tend to have different “moral weight” and are operationally more important than corresponding positive notions [137]. This goes for health and disease, pleasure and pain, but also for life and death, happiness and suffering, and for good and bad [137].

The asymmetry in moral imperative finds support in “negative utilitarianism” in ethics [138], according to which minimizing suffering has priority to maximizing happiness. With reference to Hobbes, Bernard Gert argues that “evils or harms play a much more important role in morality than goods or benefits” [139, 140]. The asymmetry also finds backing in Hans Georg Gadamer’s philosophy [141] and on the asymmetry between health and disease, where health is something given that cannot be produced or “effected” [142]. Virtue ethicists may argue that certain virtues, like compassion, are triggered by pain and suffering, but not by pleasure. It can also be claimed that avoiding pain are (near) universal ends for (human) beings [143] and that the conceptions of positive wellbeing or betterment are subjective and diverse [144].

Hence, asymmetries provide logical, conceptual, and ethical reasons for medicine to focus on negative notions, such as pain, suffering, and disease and can be used to manage the expansion of the moral imperative in medicine. This is by no means a full-fledged argument for the moral primacy of negative wellbeing to positive wellbeing. That demands a more elaborate treatment. The point here is merely to indicate that there are potential ways to manage the expansion of the moral imperative of medicine.

In sum, the traditional ways of addressing moral issues in medicine (aligning medicine with its basic concepts, with its ethos, with its basic goals, and with conceptions of making medicine better) may not work for addressing the expansion of the moral imperative in medicine. While this may seem discouraging, epistemic differences between present and future uncertainties and asymmetries in ethics between negative and positive wellbeing seem to provide resources to manage the expansion of the moral imperative in medicine. As future benefits seem less certain than present ones and as what is bad seems to be less difficult to define than what is good, it appears warranted to address present pain and suffering before future wellbeing and happiness. Accordingly, it can be argued that the moral imperative of medicine has a gradient from reducing negative to promoting positive wellbeing, and from present to future. See Fig. 3.

## Discussion

This article has identified three types of expansions of the moral imperative in medicine: (1) addressing a wide range of phenomena beyond experienced pain and suffering; (2) including future benefits in addition to present harms; (3) targeting people’s positive wellbeing in addition to negative wellbeing. It acknowledges that this moral expansion has a wide range of benefits, e.g., being able to help in new ways and increasing the power of medicine. However, it also comes at some costs. First and foremost, it extends the range of iatrogenic harms [49], such as overdiagnosis, overtreatment, medicalization, risk aversion, healthism etc. [49] and raises ethical concerns with respect to non-maleficence, beneficence, and justice, but also with the power and responsibility of professionals, as well as professional integrity and professional-beneficiary relationship. Table 2 provides a summary of the structure and the findings of this article and the many actors and drivers behind the moral expansion of medicine [145].

The analysis implies that health professionals and health policy makers gain extended responsibility, and in the case of human enhancement medicine also becomes (partly) responsible for human evolution in unprecedented way [146].

**Table 2:** Challenges and drivers of the various types of moral expansion of medicine

Expansion of moral imperative	Challenges	Drivers (Stakeholders)
1. From pain and suffering to other phenomena (indicators, ordinary-life experience, aesthetics) (*expansion of morally relevant phenomena)*	Overdiagnosis Overtreatment Risk aversion Health anxiety Healthism Ethical issues: Non-maleficence Beneficence Justice Power of professionals Responsibility Distracting responsibility Altering the professional-beneficiary relationship	People (demands, needs, preferences) Professionals (increasing knowledge, actionability, ability to help, status, prestige) Industry (tech/solutions, revenues) Media (attention, setting agenda)
2. From present to future pain and suffering (*temporal expansion*)		Law (liability, defensive medicine) Beliefs/biases (“early is better than late,” “prevention is better than cure”)
3. From negative wellbeing (pain and suffering) to positive wellbeing (*expansion of wellbeing*)		Society (“magic bullet,” perfectionism, risk aversion, ambitions, welfarism) Individuals (pursuit of positive wellbeing and happiness)

While some drivers are more specific to certain types of expansion, there is also overlap

The identified types of moral expansion are by no means absolute. They could have been classified otherwise and there are overlaps between them. For example, the expansion of disease-related phenomena is closely related to temporal expansion. When expanding the phenomena that fall under the concept of diabetes (e.g., with pre-diabetes) one also includes what can become diabetes in the future [48], i.e., a temporal expansion. Correspondingly, expanding from present to future pain and suffering, may affect present positive wellbeing, i.e., an expansion of wellbeing. Figure 3 tries to illustrate this overlap and potential interconnectedness between the types of expansion. Hence, the types of moral expansion are only one way to analyze the expansion of medicine’s moral imperative. Moreover, each of the types of expansion, and their sub-categories and examples merit deeper ethical analysis than can be presented here. The space only allows for an overview.

The phrasing “the moral expansion of medicine” (in the title) is imprecise. Even the precising phrasing in the text, i.e., “the expansion of the moral imperative of medicine” is not as precise as one could want. “The expansion of the subject matter of the moral imperative of medicine” is the more exact phrasing, but as this is quite a cumbersome expression, I have used the former expressions in order to ease the readability.

The term “medicine” is also used quite broadly. While I write about the expansion OF medicine, and describe some of the drivers thereof, medicine may appear as a unified phenomenon (with agency). Certainly, what falls under the concept of medicine may be contingent on context and vary broadly. What I refer to with medicine in the context of its moral expansion is what a broad range of competency-based institutions and practices are expected to address (morally) from the viewpoint of individuals, professionals, and society at large. Hence, the intentions of social actors may make medicine appear intentional.

Moreover, it is important to notice that this article (with the precising formulation) identifies a threefold expansion of the target of the moral imperative of medicine: new phenomena (indicators) are targeted (to help people) because they fall under the expanded concept of disease without being related to the experience of pain and suffering; there is a temporal expansion of the relevance of these phenomena (from present to future); and medicine expands its moral imperative towards positive wellbeing.

While the objective of this article has been to study *how we can manage the expansion of the moral imperative in medicine in order to obtain the benefits and avoid the harms* it has not denied that the expansion can be good. Clearly medicine has a substantial impact on the present and future positive wellbeing of people. When scrutinizing the expansion from negative to positive wellbeing, the point is not to criticize medicine’s impact on positive wellbeing. The point has been to draw attention to the negative aspects of making positive wellbeing the primary purpose or goal of medicine.

This study has only differentiated between negative and positive wellbeing. There are very many conceptions [147] and theories [148] of wellbeing, and the expansion of the moral imperative of medicine could have been investigated in terms of various hedonist, desire/preference, and objective list theories of wellbeing. While of interest for future research, that is beyond the scope of this article.

Another important topic that has not been addressed explicitly is the qualification of the term “moral imperative.” Our obligations towards other persons could range from motivation, reason, appeal, impetus, or a moral duty to act. While highly interesting and relevant, this discussion is beyond the topic of this study. Suffice it to notice that many countries have encoded a general civil duty to help persons (e.g., in terms of a “rule to rescue”) [149]. The reader may replace "moral imperative" with the preferred concept (e.g., "moral appeal"). Investigating the role various kinds of moral appeal in different areas of expansion is a relevant topic for further research.

Another interesting issue is whether the described moral expansion is much less problematic if it affects only privately paid for medical treatment. This deserves more scrutiny, but harm, lack of beneficence, lack of information/consent, distraction, power, and professional integrity may be problems within a privately funded health care system as well.

A critical reader may deny the suggested implications of the moral expansion, such as overdiagnosis, overtreatment, health anxiety, and healthism, or reject that these are negative. Alternatively, people may argue that they are inevitable (and contingent on much larger positive implications). However, the problem of showing that the benefits outrun the harms still remain [46].

Additionally, opponents may reject that there are asymmetries in ethics (making negative wellbeing morally more significant than positive wellbeing) or that future events are more uncertain than present events (epistemic differences). Here, I have only argued that these perspectives provide resources for managing the expansion of the moral imperative of medicine. Full-fledged analyses will be the topic of future studies. Certainly, there may also be other ways to manage the expansion of the moral imperative of medicine than discussed here, which I would welcome.

The examples of problems that have been used in this study are not new: overdiagnosis, overtreatment, medicalization, risk aversion, and healthism. Neither are the concerns that they raise. What is new is identifying them in the expansion of the moral imperative of medicine, i.e., in the realm of ethics, and not in the technical, behavioral, managerial, organizational or policy making realm. Failing to acknowledge the moral aspects of the fundamental challenges to modern medicine may explain their resistance to resolutions.

## Conclusion

The moral imperative of medicine is expanding in three ways. First, from targeting experienced phenomena, such as pain and suffering, to focus on non-experienced phenomena (biomarkers, risk factors, precursors, social behavior). Second, from addressing present pain to potential future suffering. Third, from negative wellbeing (pain and suffering) to positive wellbeing.

These phenomenal, temporal, and wellbeing expansions of the subject matter of the moral imperative of medicine clearly augments its capacity to help. At the same time, it extends problems such as medicalization, overdiagnosis, overtreatment, risk aversion, stigmatization, and healthism. This threatens to infringe ethical principles, such as non-maleficence, beneficence, and justice, to distract attention and responsibility from other competent agents and institutions, to enhance the power and responsibility of professionals, and to change the professional-beneficiary relationship.

Traditional approaches to address moral issues in medicine do not seem to provide sufficient guidance to manage its moral expansion. That goes for aligning medicine with its basic concepts, such as disease, therapy, and natural; to bring medicine in accordance with its ethos; or to make medicine adhere to its basic goals.

However, basic asymmetries in ethics suggest that it is more justified to address people’s negative wellbeing (pain and suffering) than their positive wellbeing. Moreover, differences in epistemology, indicate that it is less uncertain to address present pain and suffering than future wellbeing and happiness. Accordingly, if it is more difficult to define good than bad and future benefits are less certain than present, it appears warranted to address present pain and suffering before future pleasure and wellbeing. Hence, the moral imperative of medicine has a gradient from pain and suffering to wellbeing and happiness, and from present to future.

## Data Availability

All data are available in publication.

## Abbreviations

ADHD: Attention deficit/hyperactivity disorder
DCIS: Ductal carcinoma in situ
ICD: International statistical classification of diseases and related health problems
NICE: The National Institute for Health and Care Excellence
